# No flare after pre-exposure prophylaxis with monoclonal antibodies against SARS-COV2 in patients with immune-mediated rheumatic diseases: a Brazilian experience

**DOI:** 10.1186/s42358-025-00486-z

**Published:** 2025-10-24

**Authors:** Maria Fernanda Zacarin, Carolina Wonhnrath Menuzzo, Debora Pessoa De Souza, Regis Suwa Marques, Rodrigo Da Rocha Jorge, Vitor de Castro Grotti, Thiago Junqueira Trevisan, Lilian Tiemi Hirata, Marília Paula Souza dos Santos, Juliana Zonzini Gaino Borghi, Michel Alexandre Yazbek, Ana Paula Toledo Del Rio, Lilian Tereza Lavras Costallat, Zoraida Sachetto, Manoel Barros Bertolo, Alisson Pugliesi

**Affiliations:** Department of Orthopedics, Rheumatology and Traumatology, Universidade Estadual de Campinas, 126 Tessália Vieira de Camargo Street, University City, Campinas, São Paulo, 13083-887 Brazil

Dear Editor,

SARS-CoV-2 infection is associated with increased mortality in patients with immune-mediated rheumatic diseases (IMRD), particularly in those under intense immunosuppression, such as rituximab, who also show impaired vaccine responses [1]. Therefore, pre-exposure prophylaxis with monoclonal antibodies is considered for this high-risk group.

Tixagevimab/cilgavimab is the only drug developed for this purpose [2]. However, it lost efficacy against new variants and, in Brazil, its emergency authorization was suspended by ANVISA on March 7, 2023 [3]. Other monoclonal antibodies are in development, including cilgavimab-containing combinations [4]. Data on IMRD are scarce [5] and the frequency of flares after administration has not been reported.

In December 2022, our center administered Evusheld^®^ to 82 patients with IMRD following a single donation of the drug to immunocompromised individuals. 72% were women, with a mean age of 51.9 ± 12.5 years. The most common diseases were rheumatoid arthritis (30%), systemic sclerosis (21%), ANCA-associated vasculitis (17%), and systemic lupus erythematosus (17%), in addition to less frequent conditions, such as Sjögren’s disease, antisynthetase syndrome, and IgG4-related disease. The main indication was rituximab (62%); other treatments included tocilizumab, cyclophosphamide, and conventional synthetic DMARDs, and 5% of the patients were receiving prednisone > 20 mg/day.

The first evaluation was performed at a mean of 57 ± 31.8 days, with follow-up extending until June, 2023. None of the patients experienced disease flares, defined as the need for prednisone dose escalation or introduction of a new immunosuppressive therapy, as judged by the treating physician. Three mild adverse effects were observed (headache, injection-site pain, and nausea). Regarding vaccination, almost all patients received COVID-19 vaccines: 94% completed at least two doses, 76% received three or more, and approximately 70% received four or more doses. All three patients who developed COVID-19 had been vaccinated, with one receiving three, one four, and one five doses; all were on rituximab therapy. None developed severe disease. During follow-up, three patients died of causes unrelated to COVID-19.

This is the first report to describe the absence of disease reactivation in patients with IMRD after pre-exposure prophylaxis with mAbs against SARS-CoV-2. Although limited by the small sample size and the lack of a control group, our experience may help inform future prophylactic strategies. In developing new monoclonal antibodies, it is important to assess not only efficacy, but also immunologic safety, including flare risk.

## Data Availability

No datasets were generated or analysed during the current study.
